# Supplementary material for: Ultrasound Morphometry and Mean Echogenicity of Digital Flexor Tendons, Suspensory Ligament, and Accessory Ligament of Digital Deep Flexor Tendon in Gaited Horses
Source: Animals (Basel). 2023 Apr 20;13(8):1411. doi: 10.3390/ani13081411 (PMC10135043; doi:10.3390/ani13081411)
Supplement: Supplementary file 1 [file animals-13-01411-s001.zip › Table S1.pdf]

Table S1. Mean values, standard deviations, and 95% confidence interval of morphometric variables of the digital flexor tendons and ligaments of the palmar metacarpal region of 25 Mangalarga Marchador horses.

Structure	Zone	TA (mm²)	Circumference (mm)	DP Length (mm)	LM Length (mm)
**SDFT**	1	65.63 ± 6.27 (63.18 – 68.09)	32.14 ± 1.38 (31.61 – 32.68)	6.44 ± 0.56 (6.22 – 6.67)	11.59 ± 0.57 (11.37 – 11.81)
	2	61.09 ± 5.97 (58.75 – 63.43)	32.33 ± 2.00 (31.54 – 33.11)	5.76 ± 0.60 (5.52 – 5.99)	11.83 ± 0.80 (11.51 – 12.15)
	3	60.39 ± 5.54 (58.22 – 62.56)	35.21 ± 2.82 (31.10 – 36.31)	4.81 ± 0.36 (4.67 – 4.95)	12.89 ± 0.66 (12.63 – 13.14)
	4	65.51 ± 6.45 (62.98 – 68.04)	38.36 ± 2.34 (37.45 – 39.28)	4.39 ± 0.28 (4.28 – 4.50)	14.45 ± 1.07 (14.03 – 14.86)
	5	68.02 ± 5.33 (65.93 – 70.11)	42.04 ± 2.62 (41.01 – 43.06)	4.10 ± 0.50 (3.91 – 4.30)	16.82 ± 1.15 (16.37 – 17.27)
	6	80.42 ± 4.11 (78.81 – 82.03)	53.14 ± 3.17 (51.90 – 54.38)	3.63 ± 0.25 (3.53 – 3.73)	22.63 ± 0.95 (22.26 – 23.01)
**DDFT**	1	86.47 ± 7.21 (83.64 – 89.30)	37.74 ± 2.16 (36.90 – 38.59)	7.36 ± 0.33 (7.23 – 7.49)	12.63 ± 0.94 (12.26 – 13.00)
	2	74.76 ± 7.95 (71.65 – 77.88)	33.36 ± 1.94 (32.61 – 34.12)	7.41 ± 0.61 (7.17 – 7.64)	11.23 ± 0.73 (10.94 – 11.51)
	3	63.87 ± 4.95 (61.93 – 65.81)	30.08 ± 1.20 (29.61 – 30.55)	7.41 ± 0.49 (7.22 – 7.60)	10.11 ± 0.67 (9.84 – 10.37)
	4	61.99 ± 7.44 (59.08 – 64.91)	30.24 ± 1.88 (29.50 – 30.98)	7.13 ± 0.43 (6.96 – 7.30)	10.20 ± 0.78 (9.90 – 10.51)
	5	95.30 ± 6.51 (92.75 – 97.86)	36.67 ± 1.48 (36.09 – 37.25)	8.56 ± 0.38 (8.41 – 8.70)	12.90 ± 0.79 (12.59 – 13.21)
	6	116.86 ± 7.85 (113.79 – 119.94)	43.73 ± 2.03 (42.94 – 44.53)	8.01 ± 0.48 (7.82 – 8.20)	17.69 ± 1.17 (17.23 – 18.15)
**ALDDFT**	1	65.65 ± 5.38 (63.54 – 67.76)	36.19 ± 1.81 (35.47 – 36.90)	4.88 ± 0.45 (4.71 – 5.06)	13.10 ± 0.79 (12.79 – 13.42)
	2	60.53 ± 4.96 (58.59 – 62.48)	35.80 ± 1.89 (35.06 – 36.54)	4.79 ± 0.44 (4.62 – 4.97)	11.76 ± 0.87 (11.42 – 12.10)
	3	54.46 ± 3.70 (53.01 – 55.91)	36.79 ± 2.10 (35.96 – 37.61)	4.37 ± 0.61 (4.13 – 4.61)	11.29 ± 0.78 (10.98 – 11.59)
	4	49.97 ± 3.15 (48.74 – 51.20)	35.37 ± 1.88 (34.64 – 36.11)	3.92 ± 0.19 (3.84 – 4.00)	11.80 ± 0.69 (11.53 – 12.07)
**SL**	1	86.59 ± 4.27 (84.92 – 88.27)	37.59 ± 1.92 (36.84 – 38.35)	6.85 ± 0.60 (6.61 – 7.08)	12.96 ± 0.73 (12.68 – 13.24)
	2	84.06 ± 2.94 (82.91 – 85.21)	36.48 ± 1.58 (35.86 – 37.10)	7.01 ± 0.44 (6.84 – 7.18)	12.42 ± 0.77 (12.12 – 12.72)
	3	81.82 ± 3.16 (80.58 – 83.06)	35.82 ± 1.80 (35.11 – 36.52)	6.96 ± 0.53 (6.75 – 7.17)	12.31 ± 0.85 (11.98 – 12.64)
	4	81.83 ± 2.17 (80.98 – 82.68)	36.01 ± 1.63 (35.37 – 36.65)	6.82 ± 0.58 (6.59 – 7.05)	12.34 ± 0.88 (12.00 – 12.69)
**LB-SL**	1	49.39 ± 7.17 (46.58 – 52.20)	26.85 ± 1.74 (26.17 – 27.54)	6.61 ± 0.61 (6.37 – 6.85)	8.66 ± 0.86 (8.32 – 8.99)
	2	61.69 ± 6.55 (59.12 – 64.25)	29.80 ± 1.95 (29.03 – 30.57)	7.83 ± 0.59 (7.60 – 8.06)	9.36 ± 1.10 (8.93 – 9.79)
	3	101.70 ± 8.02 (98.56 – 104.85)	42.25 ± 1.98 (41.47 – 43.02)	8.09 ± 0.81 (7.77 – 8.41)	12.69 ± 0.87 (12.35 – 13.03)
**MBSL**	1	45.23 ± 5.26 (43.17 – 47.30)	25.53 ± 1.74 (24.84 – 26.21)	6.17 ± 0.50 (5.97 – 6.36)	8.42 ± 0.87 (8.08 – 8.76)
	2	61.41 ± 7.31 (58.55 – 64.28)	29.63 ± 1.87 (28.90 – 30.36)	7.36 ± 0.62 (7.12 – 7.61)	9.49 ± 0.96 (9.11 – 9.86)
	3	100.72 ± 7.77 (97.67 – 103.77)	42.85 ± 1.88 (42.11 – 43.58)	7.93 ± 0.61 (7.69 – 8.17)	12.83 ± 0.97 (12.46 – 13.21)

SDFT: superficial digital flexor tendon; DDFT: deep digital flexor tendon; ALDDFT: accessory ligament of the deep digital flexor tendon; SL: suspensory ligament; LB-SL: lateral branch of the suspensory ligament; MBSL: medial branch of the suspensory ligament; TA: transverse area; DP: dorsopalmar; LM: lateromedial.
