# Supplementary material for: Ultrasound Morphometry and Mean Echogenicity of Digital Flexor Tendons, Suspensory Ligament, and Accessory Ligament of Digital Deep Flexor Tendon in Gaited Horses
Source: Animals (Basel). 2023 Apr 20;13(8):1411. doi: 10.3390/ani13081411 (PMC10135043; doi:10.3390/ani13081411)
Supplement: Supplementary file 1 [file animals-13-01411-s001.zip › Table S2.pdf]

Table S2. Mean values, standard deviations, and 95% confidence interval of morphometric variables of the digital flexor tendons and ligaments of the plantar metatarsal region of 25 Mangalarga Marchador horses.

Structure	Zone	TA (mm^2)	Circumference (mm)	DP Length (mm)	LM Length (mm)
**SDFT**	1	62.52 ± 4.89 (60.60 – 64.43)	32.18 ± 1.56 (31.57 – 32.79)	5.66 ± 0.38 (51.51 – 5.81)	12.27 ± 0.67 (12.00 – 12.53)
	2	64.10 ± 4.35 (62.39 – 65.80)	33.01 ± 1.41 (32.45 – 33.56)	5.56 ± 0.40 (5.40 – 5.71)	12.46 ± 0.82 (12.15 – 12.76)
	3	62.55 ± 5.35 (60.45 – 64.65)	35.75 ± 3.22 (34.49 – 37.49)	4.90 ± 0.37 (4.75 – 5.04)	13.66 ± 1.14 (13.25 – 14.08)
	4	63.51 ± 5.56 (61.33 – 65.69)	38.74 ± 2.74 (37.67 – 39.81)	4.33 ± 0.36 (4.19 – 4.47)	15.09 ± 0.94 (14.76 – 15.42)
	5	65.29 ± 6.12 (62.89 – 67.69)	41.47 ± 2.35 (40.55 – 42.39)	3.90 ± 0.31 (3.78 – 4.02)	16.90 ± 0.95 (16.58 – 17.23)
	6	73.99 ± 5.78 (71.72 – 76.26)	52.84 ± 3.79 (51.35 – 54.32)	3.42 ± 0.28 (3.31 – 3.53)	22.95 ± 1.93 (22.31 – 23.59)
**DDFT**	1	83.40 ± 5.10 (81.40 – 85.40)	34.84 ± 1.22 (34.36 – 35.32)	7.77 ± 0.36 (7.63 – 7.91)	12.75 ± 0.73 (12.46 – 13.04)
	2	85.11 ± 8.26 (81.87 – 88.34)	34.67 ± 1.66 (34.02 – 35.32)	8.16 ± 0.55 (7.94 – 8.38)	12.21 ± 0.65 (11.96 – 12.46)
	3	82.57 ± 9.04 (79.02 – 86.11)	33.78 ± 1.72 (33.11 – 34.46)	8.44 ± 0.78 (8.13 – 8.74)	11.34 ± 0.56 (11.12 – 11.56)
	4	80.13 ± 9.38 (76.45 – 83.81)	33.52 ± 1.78 (32.82 – 34.21)	8.11 ± 0.52 (7.90 – 8.31)	11.28 ± 0.71 (11.00 – 11.56)
	5	98.77 ± 8.52 (95.43 – 102.11)	37.42 ± 1.76 (36.73 – 38.11)	8.77 ± 0.53 (8.56 – 8.98)	13.25 ± 0.83 (12.93 – 13.58)
	6	124.79 ± 7.20 (121.96 – 127.61)	44.82 ± 1.41 (44.27 – 45.37)	8.38 ± 0.49 (8.18 – 8.57)	17.99 ± 0.76 (17.69 – 18.28)
**ALDDFT**	1	24.15 ± 5.60 (21.96 – 26.34)	24.66 ± 3.56 (23.27 – 26.06)	2.38 ± 0.41 (2.22 – 2.55)	10.38 ± 1.49 (9.80 – 10.97)
	2	23.39 ± 5.48 (21.24 – 25.54)	23.14 ± 3.07 (21.94 – 24.35)	2.52 ± 0.36 (2.38 – 2.66)	9.64 ± 1.22 (9.16 – 10.11)
	3	21.33 ± 4.27 (19.65 – 23.00)	21.51 ± 2.09 (20.69 – 22.33)	2.57 ± 0.42 (2.41 – 2.74)	8.71 ± 0.98 (8.33 – 9.10)
	4	20.07 ± 3.15 (18.84 – 21.30)	20.79 ± 1.68 (20.14 – 21.45)	2.53 ± 0.33 (2.40 – 2.66)	8.49 ± 0.64 (8.24 – 8.74)
**SL**	1	126.26 ± 10.10 (122.30 – 130.22)	42.76 ± 2.00 (41.98 – 43.55)	9.28 ± 0.49 (9.09 – 9.47)	14.89 ± 0.87 (14.55 – 15.23)
	2	90.91 ± 7.12 (88.12 – 93.70)	36.07 ± 1.49 (35.49 – 36.66)	8.08 ± 0.41 (7.93 – 8.24)	12.48 ± 0.70 (12.21 – 12.76)
	3	90.86 ± 7.21 (88.04 – 93.69)	36.25 ± 1.63 (35.61 – 36.88)	8.05 ± 0.57 (7.82 – 8.27)	12.30 ± 0.99 (11.91 – 12.68)
	4	87.96 ± 5.87 (85.66 – 90.26)	36.23 ± 1.46 (35.66 – 36.80)	7.59 ± 0.58 (7.36 – 7.82)	12.45 ± 0.92 (12.09 – 12.81)
**LB-SL**	1	47.11 ± 4.92 (45.18 – 49.04)	25.95 ± 1.42 (25.39 – 26.51)	6.31 ± 0.58 (6.08 – 6.54)	8.62 ± 0.66 (8.36 – 8.88)
	2	60.99 ± 5.71 (58.75 – 63.23)	29.34 ± 1.35 (28.81 – 29.87)	0.76 ± 0.65 (7.31 – 7.82)	9.73 ± 0.56 (9.51 – 9.94)
	3	107.97 ± 10.12 (104.01 – 111.94)	42.82 ± 2.23 (41.94 – 43.70)	0.80 ± 0.46 (7.77 – 8.13)	13.58 ± 0.72 (13.29 – 13.86)
**MBSL**	1	43.55 ± 4.56 (41.76 – 45.34)	24.78 ± 1.30 (24.27 – 25.29)	6.17 ± 0.50 (5.97 – 6.36)	7.93 ± 0.41 (7.77 – 8.09)
	2	58.42 ± 5.18 (56.39 – 60.45)	28.84 ± 1.28 (28.34 – 29.34)	7.12 ± 0.53 (6.91 – 7.32)	9.14 ± 0.53 (8.94 – 9.35)
	3	107.59 ± 9.88 (103.72 – 111.47)	44.04 ± 2.52 (43.05 – 45.03)	7.64 ± 0.65 (7.39 – 7.90)	13.71 ± 0.64 (13.46 – 13.96)

SDFT: superficial digital flexor tendon; DDFT: deep digital flexor tendon; ALDDFT: accessory ligament of the deep digital flexor tendon; SL: suspensory ligament; LB-SL: lateral branch of the suspensory ligament; MBSL: medial branch of the suspensory ligament; TA: transverse area; DP: dorsopalmar; LM: lateromedial.
