# Supplementary material for: Ultrasound Morphometry and Mean Echogenicity of Digital Flexor Tendons, Suspensory Ligament, and Accessory Ligament of Digital Deep Flexor Tendon in Gaited Horses
Source: Animals (Basel). 2023 Apr 20;13(8):1411. doi: 10.3390/ani13081411 (PMC10135043; doi:10.3390/ani13081411)
Supplement: Supplementary file 1 [file animals-13-01411-s001.zip › Table S3.pdf]

Table S3. Mean values, standard deviations, and 95% confidence interval of morphometric variables of the digital flexor tendons and ligaments of the palmar metacarpal region of 25 Campeiro horses.

Structure	Zone	TA (mm^2)	Circumference (mm)	DP Length (mm)	LM Length (mm)
**SDFT**	1	70.30 ± 6.58 (67.72 – 72.88)	32.83 ± 1.65 (32.18 – 33.47)	6.64 ± 0.55 (6.42 – 6.86)	12.17 ± 0.66 (11.91 – 12.42)
	2	65.07 ± 6.41 (62.55 – 67.58)	32.88 ± 1.95 (32.11 – 33.64)	5.97 ± 0.67 (5.71 – 6.23)	12.16 ± 0.82 (11.84 – 12.48)
	3	62.99 ± 6.00 (60.64 – 65.34)	35.09 ± 2.41 (34.15 – 36.04)	4.92 ± 0.55 (4.71 – 5.14)	13.61 ± 1.07 (13.20 – 14.03)
	4	67.58 ± 4.68 (65.75 – 69.42)	38.63 ± 2.00 (37.84 – 39.41)	4.60 ± 0.25 (4.50 – 4.70)	15.20 ± 0.89 (14.86 – 15.55)
	5	72.09 ± 4.72 (70.25 – 73.94)	42.97 ± 1.94 (42.21 – 43.73)	4.25 ± 0.24 (4.16 – 4.35)	17.74 ± 0.81 (17.43 – 18.06)
	6	83.70 ± 3.58 (82.30 – 85.10)	55.29 ± 2.41 (54.35 – 56.24)	3.61 ± 0.17 (3.54 – 3.68)	23.72 ± 1.25 (23.23 – 24.21)
**DDFT**	1	86.87 ± 6.54 (84.31 – 89.44)	36.88 ± 2.25 (36.00 – 37.76)	7.88 ± 0.39 (7.73 – 8.03)	12.83 ± 1.01 (12.43 – 13.22)
	2	75.39 ± 6.67 (72.77 – 78.00)	32.85 ± 1.81 (32.14 – 33.56)	7.89 ± 0.66 (7.63 – 8.14)	11.25 ± 0.81 (10.93 – 11.57)
	3	65.44 ± 5.59 (63.25 – 67.63)	30.11 ± 1.99 (29.33 – 30.89)	7.96 ± 0.40 (7.80 – 8.12)	10.01 ± 0.73 (9.73 – 10.30)
	4	63.72 ± 4.86 (61.81 – 65.63)	29.49 ± 1.21 (29.02 – 29.97)	7.59 ± 0.42 (7.42 – 7.75)	10.06 ± 0.54 (9.85 – 10.26)
	5	98.77 ± 7.12 (95.97 – 101.56)	37.21 ± 1.40 (36.66 – 37.76)	8.81 ± 0.58 (8.58 – 9.04)	13.31 ± 0.68 (13.05 – 13.58)
	6	124.10 ± 8.98 (120.58 – 127.62)	44.91 ± 2.35 (43.99 – 45.83)	8.35 ± 0.36 (8.21 – 8.50)	18.27 ± 1.33 (17.75 – 18.80)
**ALDDFT**	1	65.72 ± 6.90 (63.02 – 68.43)	35.94 ± 2.07 (35.12 – 36.75)	4.99 ± 0.56 (4.77 – 5.21)	13.47 ± 1.18 (13.01 – 13.93)
	2	61.08 ± 5.97 (58.74 – 63.42)	34.95 ± 1.76 (34.27 – 35.64)	4.76 ± 0.47 (4.57 – 4.94)	12.62 ± 0.84 (12.29 – 12.95)
	3	54.79 ± 5.47 (52.65 – 56.93)	35.87 ± 2.31 (34.97 – 36.78)	4.34 ± 0.40 (4.19 – 4.50)	12.36 ± 0.80 (12.05 – 12.68)
	4	52.58 ± 5.78 (50.31 – 54.84)	36.46 ± 1.75 (35.77 – 37.14)	3.96 ± 0.44 (3.79 – 4.13)	12.58 ± 0.57 (12.36 – 12.81)
**SL**	1	90.82 ± 6.57 (88.24 – 93.39)	37.62 ± 1.57 (37.00 – 38.23)	7.29 ± 0.47 (7.11 – 7.47)	13.47 ± 0.74 (13.18 – 13.76)
	2	87.30 ± 4.49 (85.54 – 89.06)	36.14 ± 1.00 (35.75 – 36.53)	7.41 ± 0.44 (7.24 – 7.59)	12.68 ± 0.64 (12.43 – 12.93)
	3	85.04 ± 3.94 (83.49 – 86.59)	35.80 ± 1.18 (35.33 – 36.26)	7.26 ± 0.42 (7.09 – 7.42)	12.51 ± 0.68 (12.25 – 12.78)
	4	84.88 ± 3.19 (83.63 – 86.14)	35.54 ± 0.81 (35.22 – 35.86)	7.32 ± 0.31 (7.20 – 7.44)	12.45 ± 0.58 (12.22 – 12.68)
**LB-SL**	1	46.91 ± 3.18 (45.67 – 48.16)	25.29 ± 0.85 (24.96 – 25.62)	6.67 ± 0.42 (6.51 – 6.84)	8.51 ± 0.39 (8.36 – 8.66)
	2	59.48 ± 3.83 (57.99 – 60.98)	28.52 ± 0.97 (28.14 – 28.90)	7.47 ± 0.33 (7.34 – 7.60)	9.54 ± 0.52 (9.34 – 9.75)
	3	103.50 ± 7.63 (100.51 – 106.49)	40.82 ± 1.77 (40.12 – 41.51)	8.15 ± 0.36 (8.01 – 8.30)	13.08 ± 0.59 (12.85 – 13.31)
**MBSL**	1	44.21 ± 4.04 (42.63 – 45.79)	24.71 ± 1.05 (24.29 – 25.12)	6.29 ± 0.36 (6.15 – 6.43)	8.40 ± 0.44 (8.23 – 8.58)
	2	57.22 ± 4.96 (55.27 – 59.16)	28.17 ± 1.31 (27.65 – 28.68)	7.16 ± 0.41 (7.00 – 7.32)	9.50 ± 0.58 (9.27 – 9.73)
	3	105.17 ± 8.50 (101.84 – 108.50)	41.96 ± 1.85 (41.23 – 42.69)	8.00 ± 0.44 (7.83 – 8.17)	12.98 ± 0.59 (12.75 – 13.21)

SDFT: superficial digital flexor tendon; DDFT: deep digital flexor tendon; ALDDFT: accessory ligament of the deep digital flexor tendon; SL: suspensory ligament; LB-SL: lateral branch of the suspensory ligament; MBSL: medial branch of the suspensory ligament; TA: transverse area; DP: dorsopalmar; LM: lateromedial.
