# Supplementary material for: Ultrasound Morphometry and Mean Echogenicity of Digital Flexor Tendons, Suspensory Ligament, and Accessory Ligament of Digital Deep Flexor Tendon in Gaited Horses
Source: Animals (Basel). 2023 Apr 20;13(8):1411. doi: 10.3390/ani13081411 (PMC10135043; doi:10.3390/ani13081411)
Supplement: Supplementary file 1 [file animals-13-01411-s001.zip › Table S4.pdf]

Table S4. Mean values, standard deviations, and 95% confidence interval of morphometric variables of the digital flexor tendons and ligaments of the plantar metatarsal region of 25 Campeiro horses.

Structure	Zone	TA (mm²)	Circumference (mm)	DP Length (mm)	LM Length (mm)
**SDFT**	1	67.46 ± 4.69 (65.62 – 69.29)	33.65 ± 1.86 (32.93 – 34.38)	5.90 ± 0.39 (5.75 – 6.05)	13.21 ± 1.04 (12.80 – 13.61)
	2	66.77 ± 4.23 (65.11 – 68.43)	33.86 ± 1.64 (33.22 – 34.51)	5.67 ± 0.43 (5.50 – 5.84)	13.29 ± 0.94 (12.93 – 13.66)
	3	69.42 ± 4.67 (67.59 – 71.25)	37.38 ± 2.20 (36.52 – 38.24)	4.93 ± 0.41 (4.77 – 5.10)	14.66 ± 1.00 (14.27 – 15.05)
	4	68.64 ± 5.11 (66.64 – 70.64)	39.71 ± 1.75 (39.03 – 40.40)	4.43 ± 0.32 (4.31 – 4.56)	15.88 ± 0.72 (15.59 – 16.16)
	5	70.46 ± 5.57 (68.28 – 72.65)	42.40 ± 2.03 (41.61 – 43.20)	3.99 ± 0.30 (3.87 – 4.11)	17.66 ± 1.02 (17.26 – 18.06)
	6	82.26 ± 4.59 (80.46 – 84.06)	55.01 ± 3.00 (53.84 – 56.19)	3.65 ± 0.31 (3.53 – 3.77)	23.99 ± 1.43 (23.43 – 24.55)
**DDFT**	1	89.21 ± 6.87 (86.52 – 91.90)	36.07 ± 1.54 (35.47 – 36.68)	8.13 ± 0.42 (7.96 – 8.29)	13.51 ± 0.76 (13.21 – 13.81)
	2	86.60 ± 5.71 (84.37 – 88.84)	34.96 ± 1.25 (34.47 – 35.45)	8.29 ± 0.37 (8.14 – 8.43)	12.67 ± 0.65 (12.42 – 12.93)
	3	85.53 ± 4.52 (83.76 – 87.30)	34.56 ± 1.00 (34.17 – 34.95)	8.36 ± 0.52 (8.16 – 8.56)	12.13 ± 0.74 (11.84 – 12.43)
	4	83.83 ± 5.60 (81.63 – 86.02)	34.21 ± 1.21 (33.74 – 34.69)	8.26 ± 0.29 (8.14 – 8.37)	12.09 ± 0.61 (11.85 – 12.33)
	5	99.76 ± 8.61 (96.39 – 103.14)	37.59 ± 1.67 (36.94 – 38.25)	8.68 ± 0.57 (8.46 – 8.90)	13.54 ± 0.93 (13.17 – 13.91)
	6	128.09 ± 8.55 (124.74 – 131.44)	45.82 ± 2.03 (45.02 – 46.61)	8.39 ± 0.35 (8.25 – 8.53)	18.63 ± 1.11 (18.19 – 19.07)
**ALDDFT**	1	28.94 ± 7.20 (26.12 – 31.76)	27.88 ± 4.13 (26.26 – 29.50)	2.61 ± 0.52 (2.40 – 2.81)	11.51 ± 1.82 (10.80 – 12.23)
	2	25.08 ± 4.21 (23.43 – 26.73)	24.75 ± 2.85 (23.63 – 25.87)	2.50 ± 0.46 (2.32 – 2.68)	10.26 ± 1.35 (9.73 – 10.79)
	3	22.14 ± 3.32 (20.84 – 23.44)	23.18 ± 2.51 (22.20 – 24.17)	2.38 ± 0.23 (2.29 – 2.47)	9.54 ± 1.06 (9.13 – 9.96)
	4	19.71 ± 2.87 (18.58 – 20.83)	21.80 ± 1.70 (21.13 – 22.47)	2.27 ± 0.27 (2.16 – 2.38)	8.92 ± 0.83 (8.59 – 9.24)
**SL**	1	135.84 ± 8.67 (132.44 – 139.24)	44.38 ± 1.91 (43.64 – 45.13)	9.56 ± 0.36 (9.42 – 9.70)	15.89 ± 0.91 (15.53 – 16.25)
	2	87.65 ± 5.91 (85.34 – 89.97)	35.58 ± 1.44 (35.02 – 36.14)	7.83 ± 0.36 (7.69 – 7.97)	12.20 ± 0.64 (11.95 – 12.45)
	3	87.34 ± 5.20 (85.30 – 89.38)	35.80 ± 1.26 (35.30 – 36.29)	7.63 ± 0.33 (7.50 – 7.76)	12.56 ± 0.74 (12.27 – 12.85)
	4	87.06 ± 4.59 (85.26 – 88.86)	35.97 ± 1.23 (35.48 – 36.45)	7.48 ± 0.29 (7.37 – 7.59)	12.42 ± 0.60 (12.18 – 12.65)
**LB-SL**	1	46.70 ± 5.16 (44.68 – 48.73)	25.47 ± 1.34 (24.95 – 26.00)	6.63 ± 0.51 (6.43 – 6.83)	8.30 ± 0.57 (8.07 – 8.52)
	2	60.39 ± 5.01 (58.43 – 62.35)	28.79 ± 1.26 (28.30 – 29.28)	7.75 ± 0.57 (7.53 – 7.98)	9.51 ± 0.73 (9.22 – 9.80)
	3	109.07 ± 10.08 (105.11 – 113.02)	42.11 ± 2.12 (41.28 – 42.93)	8.44 ± 0.45 (8.27 – 8.62)	13.67 ± 0.69 (13.40 – 13.94)
**MBSL**	1	43.65 ± 4.30 (41.96 – 45.34)	24.76 ± 1.50 (24.17 – 25.35)	6.27 ± 0.66 (6.02 – 6.53)	8.26 ± 0.47 (8.08 – 8.44)
	2	57.39 ± 5.81 (55.11 – 59.66)	28.12 ± 1.34 (27.59 – 28.64)	7.14 ± 0.67 (6.88 – 7.41)	9.27 ± 0.57 (9.04 – 9.49)
	3	109.35 ± 6.87 (106.65 – 112.04)	42.70 ± 1.41 (42.15 – 43.26)	8.26 ± 0.46 (8.08 – 8.44)	13.34 ± 0.61 (13.10 – 13.58)

SDFT: superficial digital flexor tendon; DDFT: deep digital flexor tendon; ALDDFT: accessory ligament of the deep digital flexor tendon; SL: suspensory ligament; LB-SL: lateral branch of the suspensory ligament; MBSL: medial branch of the suspensory ligament; TA: transverse area; DP: dorsopalmar; LM: lateromedial.
